# A dual methodology employing ion-pair chromatography and built-in UV spectrophotometry for quantifying recently approved combination of mometasone and indacaterol in a novel combined metered dose inhaler: assessing the greenness, carbon footprint, blueness, and whiteness

**DOI:** 10.1186/s13065-024-01242-y

**Published:** 2024-08-03

**Authors:** Amal A. El-Masry, Ahmed Emad F. Abbas, Yomna A. Salem

**Affiliations:** 1 Department of Medicinal Chemistry, Faculty of Pharmacy, Mansoura University, Mansoura, 35516 Egypt; 2 Analytical Chemistry Department, Faculty of Pharmacy, October 6 University, 6 October City, 12585 Giza Egypt; 3 Department of Pharmaceutical Chemistry, Faculty of Pharmacy, Sinai University-Kantara Branch, Ismailia, 341636 Egypt

**Keywords:** Mometasone, Indacaterol, Ion-pair chromatography, Eco-friendly spectroscopy, Greenness blueness whiteness assessment

## Abstract

Developing analytical techniques that align with green and sustainable chemistry principles is crucial in today's scientific landscape. This work introduces two innovative approaches for the simultaneous quantification of indacaterol (IND) and mometasone (MOM), a recently approved combination therapy for chronic obstructive pulmonary disease. These methods—rapid isocratic ion pair chromatography (IPC) and UV–visible spectrophotometry—demonstrate improved environmental sustainability, cost-effectiveness, and versatility compared to existing techniques. The optimized 4-min IPC method achieved excellent resolution (retention times 2.18 ± 0.1 min for IND and 3.95 ± 0.1 min for MOM), peak symmetry, and sensitivity. It utilizes a low-cost ion pair mobile phase of acetonitrile and acidified water containing 0.025% sodium dodecyl sulfate (50:50% v/v), making it suitable for laboratories with standard chromatographic instruments. The spectrophotometric approach offers two procedures: first derivative and ratio derivative methods. These serve as simplified, low-cost alternatives for resource-limited laboratories without access to advanced instruments. Both techniques feature simplified protocols that minimize extraction and fractionation steps. Comprehensive validation confirmed outstanding accuracy (98–102%) and precision (%2 <). Sustainability assessments using ComplexGAPI, AGREE, carbon footprint, BAGI, and RGB12 tools demonstrated enhanced environmental performance compared to existing methods. The IPC and spectrophotometry methods achieved greenness scores of 0.81 and 0.85, respectively, surpassing the 0.63–0.67 range of reported techniques. Additionally, they showed lower carbon footprints of 0.035 and 0.022 kg CO2 equivalent emissions per sample, compared to 0.079–0.092 kg for conventional procedures. The application of novel "blueness" and "whiteness" concepts using BAGI and RGB12 algorithms further confirmed superior sustainability, with scores of 87.5 & 90 for blueness and 88.1 & 89.8 for whiteness. Successfully applied to quantify IND and MOM in combined capsules, this work provides a model for eco-friendly pharmaceutical analysis that maintains high analytical reliability while improving sustainability metrics.

**Supplementary Information:**

The online version contains supplementary material available at 10.1186/s13065-024-01242-y.

## Introduction

In recent years, researchers have faced the complex challenge of balancing efficacy, eco-friendliness, and practicality when developing analytical methods [1–3]. As a result, principles of white analytical chemistry (WAC) and green analytical chemistry (GAC) have gained significant attention as a means to achieve this equilibrium and contribute to a more sustainable future in quality control practices [4–6]. One such combination that calls for greener and more sustainable quality control practices is that of a novel anti-chronic obstructive pulmonary disease (anti-COPD) combination of indacaterol (IND) and mometasone (MOM).

COPD represents a significant global health challenge, ranking as the third leading cause of death worldwide after heart disease and stroke [7]. Over recent decades, COPD treatment has evolved substantially with the introduction of potent inhaled corticosteroids (ICS) and long-acting β2-adrenoceptor agonists (LABAs) [8]. Combination therapy using ICS such as MOM and LABAs like IND in a single inhaler has become a mainstay of COPD management, offering superior efficacy and improved adherence compared to ICS monotherapy [9]. Despite these therapeutic advancements, analytical methods for quantifying these drugs have lagged behind. As of 2023, only three published methods were available, each with notable limitations especially concerning long separation time, lower sensitivity, and use of high amounts of hazardous organic solvents that hinder their use in routine quality control. The first method [10] utilizes micellar liquid chromatography, which suffers from issues with column stability over prolonged use and requires careful control of mobile phase conditions like pH in addition to the requirements for specialized instrumentation, and expensive reagents that may not be accessible to all laboratories. The second method [11] uses a complex gradient elution program and 45-min column equilibration before analysis thus not amenable to high throughput analysis. Finally, the third method [12] also employs a complex gradient elution program in addition to an expensive ultra-performance liquid chromatography that is inaccessible to most laboratories. However, two additional methods were published in 2024 [13, 14], they still rely on non-green solvents and complex instrumentation, rendering them unsuitable for routine quality control practices. Thus, there is a need for a more green, simple, rapid, and cost-effective quantification technique for these critical inhaled drugs.

Our present work aims to develop two new analytical methods to quantify IND and MOM in their combined metered dose inhaler formulation that overcomes these limitations through a dual methodological approach employing ion pair chromatography (IPC) for equipped laboratories and UV–visible spectrophotometry for under-resourced laboratories. IPC is a well-established mode of high-performance liquid chromatography that allows excellent separation through the formation of ion pairs between ionizable analytes and oppositely charged ion-pairing reagents, improving compound separation and peak resolution with consistently reproducible performance and better peak shape [15–17]. Our optimized IPC method using widely available sodium dodecyl sulfate (SDS) as an ion-pair reagent enables rapid quantification in only 4 min. The short analysis time confers the advantages of high throughput, low organic solvent use, and excellent column stability for greenness and cost-effectiveness. UV-spectrophotometry serves as an excellent alternative technique given its low operational costs and suitability for laboratories with limited resources, supporting sustainability goals. By requiring only inexpensive reagents and basic equipment like cuvettes and light sources, with minimal hazardous waste generation, it aligns well with the principles of both GAC and WAC [18, 19]. Our optimized UV method uses solvent of water (80%) and ethanol (20%) as the eco-friendly solvent, offering a practical, economical, and sustainable quantification technique.

The core objectives of this study are six-fold: First, to develop an eco-friendly isocratic chromatographic analytical method for the simultaneous quantification of IND and MOM using the IPC method that offers short analysis times, high selectivity, excellent resolution, peak symmetry, and optimal linearity and sensitivity. Second, to develop a complementary green spectroscopic approach for quantifying IND and MOM to provide an easy-to-use and affordable option in laboratories lacking access to expensive chromatographic instrumentation. Third, to comprehensively assess the greenness of the proposed methods using established tools like AGREE (Analytical Greenness Metric), Complex GAPI (Complementary Green Analytical Procedure Index), and carbon footprint. This will verify enhanced environmental benefits compared to existing methods. Fourth, to introduce and apply the novel concepts of "blueness" and "whiteness" assessment using the recently introduced Blue Applicability Grade Index (BAGI) and Red–Green–Blue 12 (RGB 12) algorithms. This will demonstrate the superiority of the proposed approaches over reported methods in terms of greenness, analytical performance, cost-effectiveness, and practical utility. Fifth, to demonstrate the applicability of the developed protocols by analyzing IND and MOM in their commercially available combined dosage form. Finally, to present an exemplary model for environmentally friendly pharmaceutical analysis that does not compromise on accuracy or performance establishing a standard for sustainable quality control practices.

## Experimental

### Reagents and materials

The solvents employed were of HPLC grade, and all the compounds were of analytical reagent grade. Sodium dodecyl sulphate (SDS, 98.5%), triethylamine, trimethylamine, acetonitrile, ethanol, methanol, and orthophosphoric acid (OPA) were acquired from Sigma-Aldrich (Steinheim, Germany). Ultra-pure water was obtained from a Milli-Q water purification system (Millipore, USA) and used throughout the analysis.

### Samples

#### Reference standards

- Reference standard of MOM with lot no. M-0190223 and a purity of 99.59% was kindly gifted by Global Napi Pharmaceuticals, 6-October City, Cairo, Egypt.
- Reference standard of IND with lot no. I0009301 and a purity of 99.65%, was generously provided by Egypt’s National Organization for Drug Control and Research.

#### Pharmaceutical sample

The pharmaceutical formulation, ATECTURE breezhaler^®^ Novartis Pharma Stein AG (Switzerland), batch number BCND3 was purchased from a local pharmacy. As per the label claim, each capsule was formulated to contain 150 µg IND and 160 µg MOM.

### Apparatus

- For the liquid chromatography, separations were performed on a Perkin Elmer Series 200 system (PerkinElmer Inc., Massachusetts, USA) coupled with a Rheodyne model 7725i injector (20 μL sample loop) and a UV/VIS detector set to 259 nm. Data acquisition and processing were done using TotalChrom Workstation software. A 0.45 μm membrane filter (MilliporeSigma, Cork, Ireland) was employed for filtration of the mobile phases prior to use. Degassing was carried out in an ultrasonic bath (model SS 101 H 230, Branson Ultrasonics Corporation, USA). pH measurements were done using a P-901 pH meter (A&E Laboratory, UK).
- The spectrophotometric analysis was conducted using a Shimadzu UV-1601 PC double-beam UV–visible spectrophotometer (Shimadzu Corporation, Kyoto, Japan) equipped with a pair of matched 1 cm quartz cells. The spectrophotometer was operated using in-built UVProbe software version 2.21 with a scanning speed of 2800 nm min^−1^ (fast scan) and bandwidth of 2 nm.

### Standard solutions

Stock solutions were prepared separately for the spectrophotometric and IPC methods. 15 mg of IND and 16 mg of MOM reference standards were accurately weighed and quantitatively transferred into a 100 mL volumetric flask. The contents of the flask were dissolved in a solvent of water (80%) and ethanol (20%), sonicated for 5 min, and then diluted to volume with the same solvent to obtain stock solutions of 150 μg/mL for IND and 160 μg/mL for MOM. The stock solutions were then appropriately diluted using the same solvent (spectrophotometry) or mobile phase (IPC) as diluents to obtain working standard solutions covering the desired calibration ranges. All stock solutions were stored at 4 °C in amber-colored containers to protect them from light and were found to be stable during the study period.

### Procedures

#### IPC method chromatographic conditions

A Shim-pack cyano column (150 mm × 4.6 mm; 5 μm particle size) was used for the separation of IND and MOM at ambient temperature (25 °C). The optimized mobile phase consisted of 50% v/v acetonitrile and 50% v/v acidified deionized water containing 0.025% w/v SDS. The pH of the aqueous component was adjusted to 3.0 using a dilute OPA solution prior to mixing. The resulting mobile phase was filtered through a 0.22 μm membrane filter and ultrasonically degassed before use. A flow rate of 1 mL min^−1^ was maintained during the analysis. Analyte detection was carried out at 259 nm. Under these chromatographic conditions, efficient separation of MOM and IND was achieved within 4 min. The mobile phase was found to retain stability for two weeks during storage at room temperature.

#### Spectroscopic method

UV–Vis’s spectra of IND and MOM solutions were acquired from 200–400 nm using a solvent of water (80%) and ethanol (20%) as blank and the overlaid zero-order absorption spectra were imported into built-in spectrophotometer software (UVProbe) for further data analysis without the need for complicated software.

### Construction of calibration curves

#### IPC method

Appropriate volumes of standard drug solutions were diluted with mobile phase to yield concentrations in the range of 1–10 μg/mL for IND and MOM. The solutions were subsequently examined by injection into the chromatographic system and eluting them using the mobile phase while adhering to the aforementioned chromatographic parameters. The calibration charts were generated by the process of plotting the peak area against the relevant concentrations, followed by the calculation of linear regression equations.

#### Spectroscopic method

A series of 10-mL volumetric flasks were prepared with aliquots of standard IND and MOM solutions ranging from 1.1–32 μg/mL for Mom and 1–30 μg/mL for IND, then filled to the required volume with a solvent of water (80%) and ethanol (20%). The absorption spectra of the prepared drug solutions were measured over 200–400 nm using this solvent as a blank.

##### First derivative method (^1^D)

The stored spectral data was processed to obtain the ^1^D spectra using the built-in UV-Probe software with wavelength difference (Δλ) = 20 nm and scaling factor (SF) = 10. The peak amplitudes at 251.62 nm for IND and 275.67 nm for MOM were measured. Calibration curves were constructed by plotting the peak amplitudes versus the corresponding concentrations in μg/mL and the relevant regression equation was calculated.

##### Ratio derivative method (RD)

Using the built-in UV-Probe software, the ratio spectra of MOM are acquired by dividing the recorded MOM's zero-order absorption spectra by the IND divisor spectrum (1 μg/mL). Similarly, the ratio spectra of IND are acquired by dividing the recorded IND's zero-order absorption spectra by the MOM divisor spectrum (1 μg/mL). The obtained ratio spectra were processed to obtain the first derivative of the ratio spectra (^1^DD) with (Δλ) = 20 nm and (SF) = 100. The peak amplitudes at 310.49 nm for IND and 280.64 nm for MOM, respectively, were measured. Calibration curves were constructed by plotting the peak amplitudes versus the corresponding concentrations in μg/mL and the relevant regression equation was calculated.

#### Analysis of laboratory-prepared mixtures

- IPC method

Aliquots of IND and MOM keeping the pharmaceutical ratio of 1:1.06 were added to 10 mL volumetric flasks, which were then completely filled with mobile phase to yield different concentration ratios of the two drugs covering the linearity range. The solutions were mixed well and analyzed under optimized IPC conditions. The concentrations of IND and MOM were determined from respective regression equations.

- Spectroscopic method

Aliquots of IND and MOM keeping the pharmaceutical ratio were added to 10 mL volumetric flasks, which were then completely filled with a solvent of water (80%) and ethanol (20%) to yield different concentration ratios of the two drugs covering the linearity range. The absorption spectra of these laboratory mixtures were recorded as described earlier. Concentrations of IND and MOM were determined using the respective spectroscopic methods.

#### Analysis of the studied drugs in their co-formulated inhaler capsule

Twenty Atectura Breezhaler^®^ inhaler capsules (labeled to contain 150 μg of IND and 160 μg of MOM per capsule) were emptied and mixed thoroughly. A precisely measured amount of powder, equal to the weight of ten capsules, was placed into a volumetric flask with a capacity of 100 mL. Approximately 50 mL of a solvent of water (80%) and ethanol (20%) was added and sonicated for 15 min to extract the drugs into the solution. The content was mixed well, passed through a membrane filter with a pore size of 0.45 μm, and appropriately diluted with the same solvent to the mark. Suitable aliquots were transferred from the clear filtrate into 10 mL volumetric flasks and diluted with the same solvent (spectrophotometry) or mobile phase (IPC). The steps were carried out in accordance with the previously outlined procedures to ascertain the concentration of the two medicines by employing the regression equations.

## Results and discussion

**Fig. 1:**
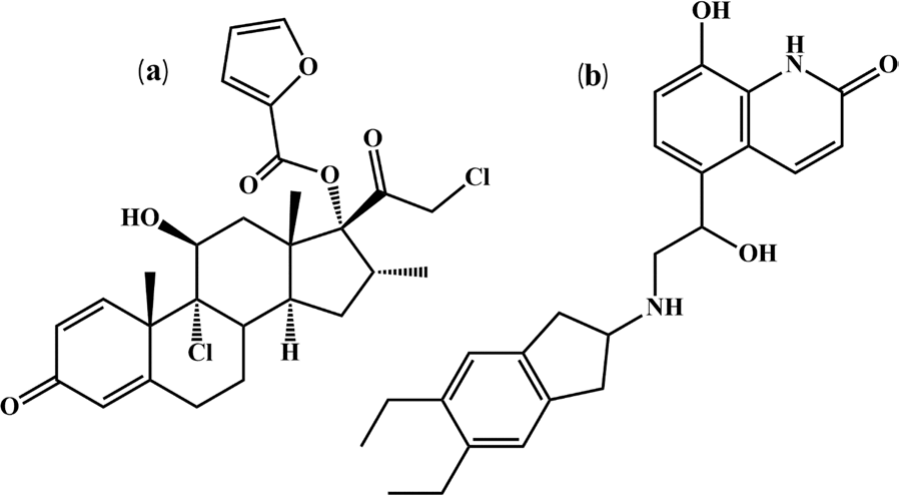
Chemical structure of **a** MOM and **b** IND

**Fig. 2:**
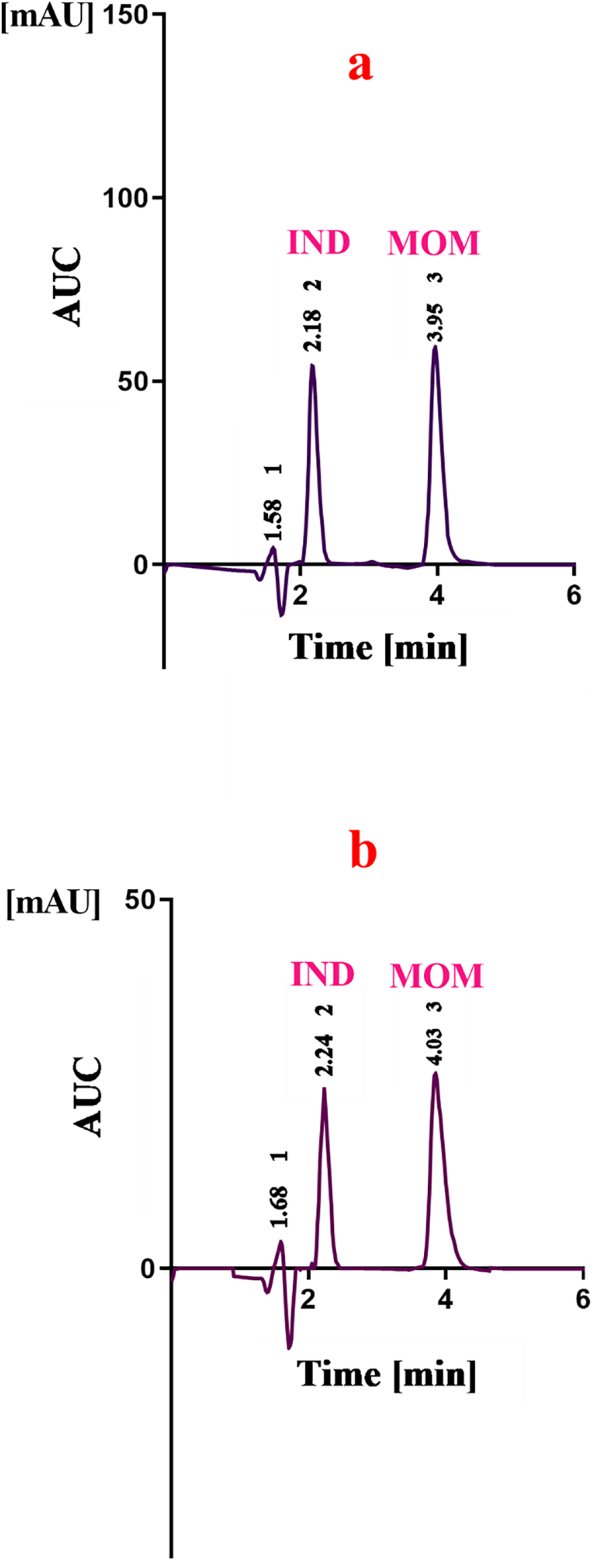
IPC chromatogram demonstrating the separation of IND and MOM. **a** IND (7.5 μg/mL, 2.18 min) and MOM (8.0 μg/mL, 3.95 min) in the synthetic mixture, **b** IND (1.5 μg/mL, 2.24 min) MOM (1.6 μg/mL, 4.03 min) in the inhaled capsule

In the current study, the approach was to produce a more effective simultaneous determination of anti-COPD medicines, IND, and MOM (Fig. 1) by employing IPC and complementary spectroscopic methods with higher sensitivity, low cost, and minimum use of hazard solvents that offer applicable methods for routine quality control analysis. Furthermore, a variety of complementary sustainability assessment techniques were used to test the presented methods' green and white attributes. The outcomes demonstrated how well the recommended approach performed according to the sustainable analytical chemistry parameter values. The developed IPC method provides efficient isocratic separation between IND and MOM within a lower elution time of only 4 min in synthetic mixtures and co-formulated metered dose inhalers, as shown in Fig. 2, which provides numerous sustainability advantages including 1—higher sample throughput that maximizes information output while minimizing overall run costs and energy consumption. 2—less solvent usage that reduces chemical waste generation and disposal burdens. 3—enhanced durability as rapid quantifications extend column lifespan and periods between maintenance, that resilience enables longer operational periods and replacement cycle times. 4—lower energy necessary and less electricity and resources are demanded for the same analytical workload. 5—substitution of organic solvents by alternative ion pairing compounds minimizes hazardous waste and toxicity risks. In the proposed method the SDS was chosen as the ideal ion pairing agent after evaluation of several options. SDS offered unique advantages including enhanced resolution and separation between IND and MOM based on altered hydrophobicity and formation of neutral ion pairs with drugs functional groups, improvement in peak shape and symmetry by controlling undesirable peak broadness and tailing, excellent column efficiency and stabilization of column performance after prolonged use, low concentration sufficient for effect (0.025% v/v) which aligns method with green chemistry principles, environmentally-friendly and less toxic compared to other conventional ion pairing agents. Additionally, the complementary spectrophotometric techniques provide a greener, more cost-effective option for quantitation in resource-limited settings, with the sustainability metrics validating their positive environmental profile.

### IPC method development and optimization

All critical chromatographic parameters were systematically optimized to obtain adequate resolution, symmetric peak shapes, and short analysis time. The optimized conditions are compiled in Table S1. The USP guidelines [20] include specific instructions for measuring chromatographic performance, including the number of theoretical plates and the resolution. The results of these measurements are provided in Table S1.

#### Selection of column

Different stationary phases were evaluated to find the optimal column that provides good separation and peak shape for IND and MOM. The columns tested included: Zorbax SB C18 column (150 × 4.6 mm, 5 μm)—This reverse phase column provided poor peak shape and co-elution of the analytes, Zorbax Eclipse XDB C8 column (150 × 4.6 mm, 5 μm)—The peak for IND was asymmetric using this column. The run time was also longer compared to other columns over 12 min, Luna C18 column (150 × 4.6 mm, 5 μm)—Partial separation of IND and MOM occurred but peaks were broad, and the total run time was over 10 min. Shim-pack cyano column (150 mm × 4.6 mm, 5 μm)—This column provided good peak symmetry for both analytes and baseline separation within only 4 min.

#### Selection of detection wavelength

Various wavelengths, including 200–300 nm, were tested. Based on spectral evaluation and a better baseline, 259 nm was selected for detection allowing high sensitivity for both drugs.

#### Optimization of mobile phase

The use of an ion pairing mobile phase offers numerous advantages over conventional organic solvents. They serve to improve resolution between analytes with similar physicochemical properties. The added ions in the agent non-covalently interact with ionized sites on the analytes and neutralize charges, altering the overall hydrophobicity and hydrophilic balance. This facilitates the separation of the ion pairs on common reverse-phase packing materials based on their new relative hydrophobicity. Various compounds with amine or other ionizable groups can be explored as possible ion-pairing agents for optimal selectivity and resolution [21–24].

Numerous mobile phase compositions were systematically assessed. Initial trials focused on three organic solvents (methanol, ethanol, and acetonitrile) in combination with three different ion-pairing agents in acidified water (triethylamine, trimethylamine, and SDS). These components were mixed at organic solvent: aqueous ratios of 40: 60, 45:55, 50:50, 55:45, and 60:40 v/v. When ethanol and methanol were used, no separation occurred for the IND and MOM. Moreover, when triethylamine and trimethylamine were used gave a wide peak with a long retention time. Ultimately acetonitrile and acidified water containing SDS (50: 50% v/v) yielded satisfactory resolution and a symmetrical peak with good clarity in sufficient time (4 min), as shown in Table S1 and Fig. S1.

#### Effect of pH and buffer concentration

The effect of pH on the chromatographic behavior of IND and MOM was studied using an increasing amount of OPA between pH 2.5 to 6, as shown in Fig. S2. Additionally, the ionic strength of OPA (M) was studied between 0.05 and 0.3 M, as shown in Fig. S3. pH 3 and 0.1 M OPA provided maximum theoretical plates and peak symmetry with well-resolved and sharp peak shapes as can be seen in Table S1.

#### Effect of concentration of SDS

Varying SDS concentrations between 0.015–0.03% w/v were assessed. Lower SDS levels of around 0.015% resulted in inadequate separation and peak broadening. Higher concentrations around 0.03% provided only marginal improvements in resolution. Ultimately, 0.025% SDS was determined to be the optimal concentration, providing excellent peak shape and symmetry in a rapid 4-min isocratic run.

#### Effect of flow rate and column temperature

The flow rate varied from 0.8 to 1.2 mL/min, as shown in Fig. S4. Optimal separation with a significant reduction in retention time was achieved at a 1 mL min^−1^ flow rate. Similarly, the column oven temperature was varied from 25° to 50 °C. While higher temperatures increased theoretical plates, the tailing was also proportionately increased. Hence, ambient temperature (25 °C) was maintained giving the best peak shapes and resolution.

### Spectrophotometric methods

**Fig. 3:**
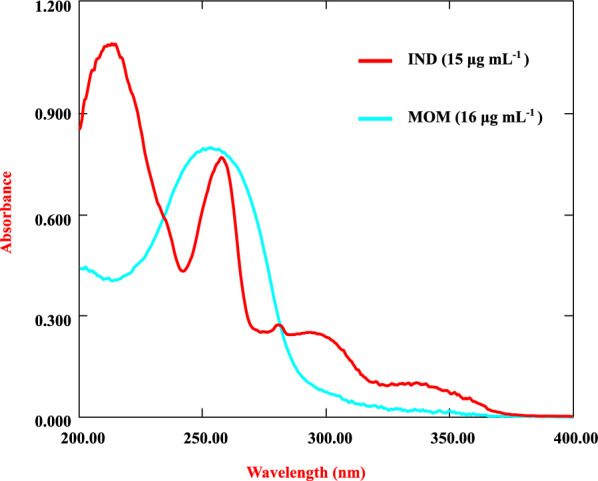
Zero order spectra of IND and MOM in a solvent of water (80%) and ethanol (20%) as blank

The overlaid UV zero-order absorption spectra of IND and MOM (Fig. 3) showed a lot of overlap, making their direct simultaneous estimation difficult. Hence, advanced spectroscopic techniques were developed for their quantification. Two simple, economical, and accurate derivative and ratio derivative methods were developed.

#### Development and optimization

##### Selection of solvent

The selection of an appropriate solvent is crucial for developing an effective and environmentally friendly spectrophotometric method. We conducted a comprehensive evaluation of various solvents to optimize the solubility and absorption intensity of IND and MOM. Our investigation encompassed pure solvents including distilled water, ethanol, acetonitrile, and methanol, as well as aqueous buffers such as 0.1 M HCl, pH 8 borate buffer, 0.1 M NaOH, and pH 4 acetate buffer. After extensive testing, we determined that a mixture of 80% water and 20% ethanol provided the optimal balance of solubility and absorption intensity for both drugs. This solvent combination demonstrated superior performance compared to other tested options, yielding maximum solubility and the highest absorption intensity for IND and MOM. The chosen solvent mixture aligns well with green chemistry principles by minimizing the use of organic solvents while maintaining excellent analytical performance. The high water content reduces environmental impact and safety concerns associated with organic solvents, while the small ethanol fraction ensures adequate solubility of the analytes. Furthermore, this solvent system is compatible with standard laboratory equipment, making it suitable for widespread adoption in various analytical settings. Its simplicity in preparation and stability during analysis contribute to the method's reproducibility and robustness. Overall, by selecting this water–ethanol mixture, we have developed a method that not only provides optimal analytical performance but also supports sustainable practices in pharmaceutical analysis. This choice reflects our commitment to balancing scientific rigor with environmental responsibility in analytical chemistry.

##### Delta lambda and scaling factor for methods

For derivative spectrophotometry, the derivative order, Δλ, and SF were optimized to give sufficient sensitivity and selectivity at working concentrations. Δλ = 20 nm and SF = 10 For the ^1^D method, and Δλ = 20 nm and SF = 100 for RD gave well-defined peaks with the best signal-to-noise ratio.

##### Selection of divisors

Different concentrations of MOM and IND standards were evaluated as divisors to obtain the optimized RD method. Concentrations of 1 μg/mL of IND and 1 μg/mL of MOM gave the best recovery with the highest sensitivity and reproducibility.

#### Method characteristics

##### First derivative method

**Fig. 4:**
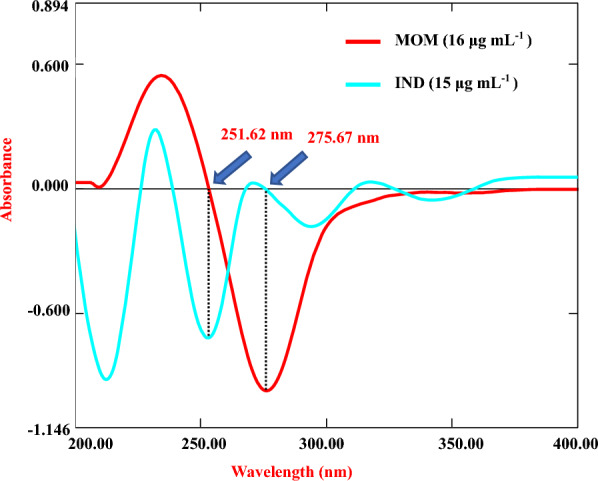
First-order derivative spectra of IND and MOM where MOM measured at 275.67 nm (at zero crossing for IND) and IND measured at 251.62 nm (at zero crossing for MOM)

This technique helps remove spectral interference by transforming the normal zero-order absorption spectra into ^1^D signals. It takes advantage of the basic principle that derivative amplitudes of a component are directly proportional to its concentration, while the influence of interfering drugs and excipients is effectively abolished [25]. The critical aspect is that each ^1^D spectrum displays unique zero-crossing points where the interference drug has no contribution. In the present work, ^1^D spectra of IND and MOM were obtained by using Δλ = 20 nm and SF = 10. For IND, the peak amplitude was selectively measured at its zero-crossing wavelength of 251.62 nm. Similarly, for MOM, quantification was performed at 275.67 nm, as shown in Fig. 4. The spectra were found to follow Beer’s law very well over the therapeutic concentration ranges. Key attributes of this method are its rapid analysis speed, simple procedure, sensitivity, cost-effectiveness, and avoidance of extensive separation steps—aligning excellently with GAC and WAC principles.

##### Ratio derivative method

**Fig. 5:**
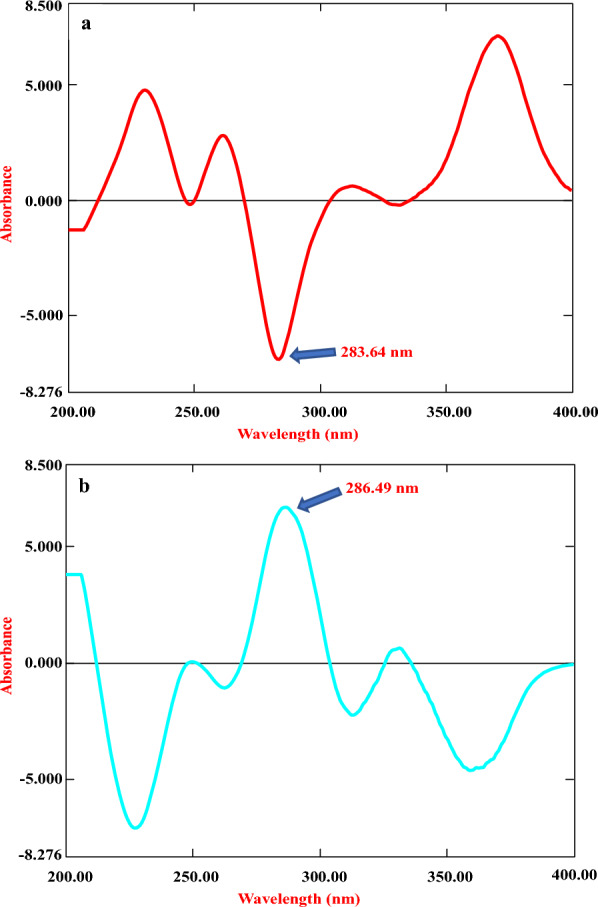
**a** First derivative of the ratio spectra of MOM when 1 μg/mL IND was used as the divisor and **b** first derivative of the ratio spectra of IND when 1 μg/mL MOM was used as the divisor

In addition to the traditional first derivative technique, the RD UV spectrophotometry method was developed for resolving overlapping absorption spectra of the IND and MOM combination [26]. RD approach entails dividing the absorption spectrum of IND or MOM (analyte) by a constant absorption spectrum of MOM or IND (divisor), respectively. The constant divisor spectrum used was 1 μg/mL for each drug. These vectorially divided analyte/divisor ratio spectra were then derivatized (^1^D) using a Δλ interval of 20 nm and a scaling factor of 100. In the resulting first derivative of the ratio spectra, well-defined peaks were observed for IND at 286.49 nm and MOM at 283.64 nm with zero crossing points ideal for analytical measurement, as shown in Fig. 5. At these wavelengths, only one particular drug component contributes such that simultaneous quantification of both is enabled without spectral interference. Calibration curves were constructed by plotting peak amplitudes versus IND or MOM concentrations. The regression equations exhibited excellent correlation coefficients (> 0.999), supporting the ratio derivative technique for selective and accurate quantification well within the therapeutic ranges. Key benefits of the developed method include its simplicity, precision, accuracy, and avoidance of tedious procedures for removing background signals from excipients and dosing vehicles. The RD approach provided a rapid, economic, and green analytical strategy aligning with GAC and WAC principles.

### Method validation

The proposed IPC and spectroscopic methods were validated as per the ICH recommendations [27].

#### System suitability testing

To evaluate system performance, system suitability parameters like the number of theoretical plates (N), Tailing factor (T), and resolution (R_s_) were all investigated as system appropriateness criteria. The optimized method provided good baseline separation of the two drugs with minimal tailing, as indicated in Table S1.

#### Linearity range

Calibration curves were constructed covering suitable wide concentration ranges encompassing therapeutic doses of the IND and MOM combination. The IPC method demonstrated excellent linear responses across 0.1–10 μg/mL for both IND and MOM. The regression statistics gave very high correlation coefficients (r > 0.999). Similarly, for the spectral techniques, the linearity range extended from 1–30 μg/mL for IND and 1.1–32 μg/mL for MOM. Again, the calibration curves displayed regression coefficients greater than 0.999, supporting the strong linear relationships. The calculated regression characteristics are summarized in Table 1. The data were analyzed statistically giving small values of the standard deviation of residuals (Sy/x), intercepts (Sa), slopes (Sb), Percentage relative standard deviation (%RDS), and Percentage relative error (% E), as shown in Table 1.

**Table 1:** The Regression parameters and validation results for determining IND and MOM by the proposed methods

Parameter	IPC method		Spectroscopic methods			
			First derivative (^1^D)		Ratio derivative (RD)	
	MOM	IND	MOM	IND	MOM	IND
Wavelength (nm)	259	259	275.67	251.62	280.64	310.49
Linearity range (µg mL^−1^)	0.1–10	0.1–10	1.1–32	1–30	1.1–32	1–30
Slope	62.347	55.128	− 0.007	0.01	0.065	0.083
Intercept	4.120	36.436	− 0.016	0.013	0.024	− 0.017
correlation coefficients (r)	0.9997	0.9998	0.9996	0.9998	0.9998	0.9999
Accuracy (%R)	99.97	99.91	100.02	99.69	100.28	100.11
Precision (%RSD)						
Repeatability^a^	0.531	0.832	0.366	0.774	0.682	0.755
Intermediate precision^b^	0.732	0.965	0.348	0.675	0.985	0.857
LOD (μg/mL)	0.026	0.031	0.290	0.314	0.202	0.227
LOQ (μg/mL)	0.079	0.094	0.879	0.951	0.612	0.688
S_y/x_	0.965	1.012	9.1 × 10^–4^	1.2 × 10^–5^	5.6 × 10^–4^	8.12 × 10^–4^
S_a_	0.5	0.52	2.0 × 10^–5^	5.00 × 10^–4^	1.0 × 10^–5^	0.01
S_b_	0.099	0.103	4.0 × 10^–5^	5.00 × 10^–5^	2.2 × 10^–5^	3.4 × 10^–5^
% RSD^c^	1.334	0.677	0.737	0.629	0.556	0.97
% Error^d^	0.471	0.239	0.301	0.257	0.227	0.395
Robustness^e^ (%RSD)	0.317	0.586	0.432	0.602	0.552	0.499

^a^The intraday (n = 3), average of three responses of three different concentrations repeated three times on the same day

^b^The interday (n = 3), average of three responses of different three concentrations repeated three times at three successive days

^c^Percentage relative standard deviation(SD × 100/mean %R)

^d^Percentage relative error. = % RSD/$\sqrt{n}$

^e^Average of three determinations

#### Sensitivity

The limits of detection (LOD) and quantification (LOQ) were determined using the slope and standard deviation of the intercepts of the regression lines as per ICH recommendations.

$$LOQ=\frac{{S}_{a}}{b}\times 10 \quad LOD=\frac{{S}_{a}}{b}\times 3.3$$

The low LOD and LOQ values indicate adequate sensitivity of the proposed methods to precisely detect and quantify IND and MOM, as shown in Table 1.

#### Accuracy and precision

To assess the method's accuracy, nine distinct measurements were made at three various concentration levels. in the IPC method, (1, 4, and 8 μg/mL) for each drug, while spectroscopic methods used (5, 15, and 25 µg mL^−1^) for each drug. The excellent percentage recoveries (%R) between 98 and 102% for repeated measurements displayed outstanding accuracy, as shown in Table 1.

Likewise for precision, standard deviations (SD) and percentage relative standard deviations (%RSD) were calculated based on replicate analyses at each concentration either on the same day (repeatability) or over three days (intermediate precision). The very low SD (< 2%) and %RSD values (< 2%) highlighted the high degree of reproducibility and low variability around the mean, as shown in Table 1.

#### Selectivity

By testing various laboratory-prepared mixtures containing IND and MOM within the linearity range, the selectivity of the techniques was determined, and promising findings were achieved. Acceptable results were achieved, as shown in Table S2.

#### Robustness

For the IPC method, the method's robustness was assessed by investigating the impact of slight modifications in pH (3.0 ± 0.2), proportion of acetonitrile (50 ± 2% v/v), and molar concentration of OPA (0.1 ± 0.02% w/v). For spectroscopic methods, robustness was examined by slightly altering experimental variables including wavelength interval (0.9 nm instead of 1 nm), spectral bandwidth (1.8 nm instead of 2 nm slit width), and Scan speed (medium instead of fast scan). No discernible impact was found in IPC and spectroscopic methods, as shown in Table 1.

#### Application to tablet preparation

Using the provided techniques, the drug content of ATECTURE breezhaler^®^ capsules was successfully estimated. The results met expectations and showed excellent accuracy with the amount claimed on the label, with no evidence of excipients interfering. Additionally, using t- and F-values with a 95% confidence level to compare the results with those from the reported methodology [10] statistically, there were no appreciable differences among the suggested and reported methods, as shown in Table S3.

## Comprehensive greenness, blueness, and whiteness evaluation

Assessing the sustainability of analytical methods requires evaluating their environmental, economic, and performance impacts across multiple criteria. No single tool can comprehensively assess all relevant parameters [28, 29]. Therefore, this work employs a combined multi-tool approach for a more holistic evaluation from different complementary perspectives.

### Evaluation of the methods' green performance based on ComplexGAPI criteria.

The innovative semi-quantitative ComplexGAPI tool enables expanded assessment of green analytical procedure impacts beyond just the final analysis step. This tool has gained significant recognition and acceptance within the chemical society due to its ability to enhance the existing GAPI metric by incorporating a supplementary hexagonal field, based on the CHEM21 parameters. This extended evaluation encompasses all stages of the analytical method, such as sample collection, preservation, transportation, sample preparation, storage, and final analysis [30], as shown in Fig. S5. The user-friendly shareware software generates a color-coded pictogram visually summarizing the greenness at each stage on a scale from green to yellow to red. It also calculates a cumulative E-factor indicating overall waste generation. Our developed methods demonstrated excellent greenness as shown by the predominance of green icons and a very low E-factor of 1, reflecting the minimal environmental burden as summarized in Table 2. However, ComplexGAPI remains limited to primarily environmental criteria. Other key dimensions like waste prevention, energy efficiency, and renewable materials usage are not addressed. Thus, combining ComplexGAPI with additional quantitative tools is recommended for a more holistic assessment.

**Table 2:** Comparative study of the proposed and reported methods

Parameter	Proposed IPC method		Reported method [10]		Reported method [11]		Reported method [12]		Proposed Spectroscopic methods	
Run time (minute)	4		8		10		7		–	
Type of elution	Isocratic		Isocratic		Gradient		Gradient		–	
Stationary phase	Shim-pack cyano column (150 mm × 4.6 mm; 5 μm particle size)		Symmetry C18 column (100 mm × 4.6 mm × 3.5 μm)		Inertsil ODS C18 column (250 × 4.6 mm, 5 µm)		Kinetex 1.7 μm HILIC 100A column (100 × 2.1 mm, 1.7 μm particle size)		–	
Mobile phase	Acetonitrile and acidified water containing 0.025% SDS as ion pair reagent (50: 50% v/v)		Methanol: micellar reagent (191.49 mM) and (10.00 mM) potassium dihydrogen phosphate buffer (22.50: 77.50 v/v),		Methanol: 0.1% glacial acetic acid (40:60, v/v), then methanol: 0.1% glacial acetic acid (85:15, v/v)		Methanol: 0.1% formic acid in water (20:80, v/v), then methanol: 0.1% formic acid in water (80:20, v/v)		–	
Wavelength	259 nm		210 nm		233 nm		220 nm		–	
Linearity range (μg/mL)	**MOM**	**IND**	**MOM**	**IND**	**MOM**	**IND**	**MOM**	**IND**	**MOM**	**IND**
	0.1–10	0.1–10	10–100	10–100	50–300	50–300	1–150	0.5–100	1.1–32	1–30
LOD (μg/mL)	**MOM**	**IND**	**MOM**	**IND**	**MOM**	**IND**	**MOM**	**IND**	**MOM**	**IND**
	0.026	0.031	2.39	2.67	4.32	7.29	0.1	0.03	0.202	0.227
Complex GAPI tool										
AGREE tool										
Carbon footprint (kg CO_2_ eq /sample)										
BAGI tool										
RGB 12 algorithm										

### Assessment of the methods' green performance based on AGREE criteria

AGREE tool enables comprehensive evaluation across all 12 principles of GAC. Its flexibility, clear visual outputs, and freely available software have promoted widespread adoption [31, 32]. AGREE allows customized weighting of parameters and scoring from 0 to 1, where 1 reflects full alignment with GAC principles. The overall result is depicted on a clock-like graph, with the center color and score indicating greenness, as shown in Fig. S5 to Fig. S10. Extensive AGREE assessments were performed for both the developed methods and the reported techniques. As shown in Table 2, the proposed approaches demonstrated outstanding greenness, achieving high scores of 0.7 (IPC) and 0.75 (Spectrophotometry) versus just 0.66, 0.63, and 0.64 for prior methods. The vivid green shading in the pictograms confirms the significant improvements in parameters like energy utilization, waste prevention, chemical safety, and use of renewable materials. This reinforces the eco-friendly nature of the strategies. However, AGREE predominantly focuses on environmental aspects of green chemistry. Critical sustainability dimensions like economic feasibility, analytical performance, and robustness are not captured. Thus, pairing AGREE with complementary tools is recommended for a more holistic evaluation, avoiding overemphasis on just green metrics.

### Carbon footprint analysis

Carbon footprint analysis provides a quantitative evaluation of analytical methods' environmental impacts based on greenhouse gas emissions, reported as kilograms of CO_2_ equivalent (kg CO_2_ eq) [33]. By accounting for electricity, transportation, materials usage, and waste, it effectively complements other green chemistry tools lacking emissions quantification. Carbon footprints were calculated using the standardized equation [34]:

$$\text{Carbon footprint}(\text{kg CO}_2 \,\text{eq})=\sum Instrument \,Power \left(\text{kW}\right) . Analysis \,time \left(\text{h}\right) . Emission \,factor (\text{kg CO}2/\text{kWh})$$

As shown in Table 2, the proposed IPC method demonstrated a markedly lower carbon footprint of just 0.035 kg CO_2_ eq versus 0.079–0.092 kg CO_2_ eq for reported techniques. This over 50% reduction reflects excellent environmental performance. Similarly, the spectrophotometry method showed the lowest emissions of 0.002 kg CO_2_ eq per sample analyzed. This substantial decrease relative to prior reports is owed to the very short analysis times cutting electricity demands combined with the simplified protocols replacing organic solvents with water and ion pairing agents markedly decreased related emissions. In summary, carbon footprint analysis confirmed the favorable green credentials of the developed methods, with lower greenhouse gas emissions demonstrating their positive sustainability profile. The quantitative results reinforce conclusions from ComplexGAPI and AGREE-based evaluations regarding the techniques’ eco-friendly nature.

### Assessment of methods' blueness based on BAGI criteria

BAGI tool enables quantitative evaluation of analytical methods’ functionality and real-world applicability (blueness) [35]. BAGI allows scoring across 10 practical criteria: analysis type, number of analytes detected, instrumentation requirements, sample throughput, preparation needs, analysis rate, reagents/materials consumed, preconcentration requirements, automation potential, and sample quantity needed. Each parameter is graded from 1 (worst) to 10 (best), as shown in Table S4. The composite BAGI index is computed as the geometric [35]. The developed IPC and spectrophotometry methods obtained high BAGI scores of 87.5 and 90 respectively, confirming excellent practicality. The rapid analysis times below 4 min for both drugs using simple equipment and procedures offer significant real-world advantages. Moreover, the eco-friendly ion pair reagent minimizes material inputs and waste generation, echoing green analytical principles within an applied context. Hence, the BAGI assessment verifies the outstanding blueness of the techniques as implementable alternatives to replace conventional methods. However, since BAGI specifically targets functional criteria, it does not provide a holistic sustainability measure. Therefore, we additionally employed the RGB12 algorithm to evaluate the composite analytical sustainability considering greenness, performance, and practicality. The consistently high scores across tools verify this method's merit as an implementable green chemistry alternative.

### Evaluation of the methods' whiteness profile

While tools like ComplexGAPI, AGREE, and BAGI provide assessments of specific sustainability aspects, the RGB12 algorithm enables quantitative evaluation of the composite analytical sustainability through whiteness measurement [36, 37]. RGB12 comprises 12 distinct components divided into red, green, and blue groups addressing parameters related to analytical validation, eco-friendliness, and practical feasibility respectively. The green group (G1–G4) focuses on important parameters such as toxicity, excessive waste and reagent utilization, energy utilization, and the immediate effects on animals, humans, and genetically modified organisms. The group denoted by the color red (R1–R4) conducts an assessment of validation criteria, encompassing the extent of applicability, the LOD, LOQ, precision, and accuracy. The cluster denoted as the blue group, comprising B1 to B4, evaluates the affordability, time-based efficiency, as well as practical and financial prerequisites. By applying the RGB algorithm, the whiteness value, which represents the method's compliance with the ideas of WAC, is estimated by combining the scores obtained for each of the three-color groups. As shown in Table 2 and Fig. S11, the developed methods attained excellent whiteness values of 88.1 (IPC) and 89.8 (Spectrophotometry). This significantly exceeds reported techniques, confirming superior alignment with sustainability principles like waste minimization, energy efficiency, precision, affordability, and practicality. The high scores reinforce conclusions from prior focused Greenness (ComplexGAPI, AGREE) and Blueness (BAGI) evaluations regarding the favorable environmental and functional profile. Additionally, the outstanding red metric performance verifies excellent analytical validation. In contrast to other narrowly-focused tools, RGB12 delivers a big-picture quantification of sustainability. Using RGB12 alongside specialized gauges for greenness, blueness provided a robust, multidimensional assessment that avoids biases from overemphasizing any single aspect like cost or precision. This systems-thinking approach applying multiple complementary evaluation tools enables a comprehensive, unbiased assessment of alignment with sustainable analytical chemistry ideals. The consistently high scores validate the developed methods as greener, cost-effective alternatives with comparable performance for wide-ranging applications.

## Conclusion

The work presented in this study successfully developed and validated three novel analytical approaches including IPC and two spectrophotometric techniques for rapid, simultaneous quantification of the anti-COPD drug combination—IND and MOM in pure and capsule preparations. Methods optimization and thorough validation as per ICH guidelines demonstrated excellent sensitivity, precision, accuracy, and linearity while analysis of lab-prepared mixtures and pharmaceutical capsules proved specificity and reliability of results for routine quality control. The proposed methods offer numerous advantages over earlier reports including straightforward protocols without elaborate extraction, and fractionation steps; inexpensive reagents and equipment; eco-friendly excluding toxic organics; very rapid analysis times; and excellent detection limits aligning with therapeutic concentrations administered by oral inhalation. Additionally, the developed approaches excel regarding waste minimization, cost-effectiveness, safety, and energy efficiency thereby excellently addressing green analytical and sustainable white analytical chemistry goals. Holistic sustainability assessment using tools like ComplexGAPI, AGREE, carbon footprint, BAGI, and RGB12 verified outstanding greenness, blueness, and whiteness metrics alongside analytical reliability and practical functionality. Thus, this work contributes to a sustainable future for analytical science by exemplifying benign practices that reconcile environmental and economic aspirations.

### Supplementary Information

Supplementary Material 1.

## Data Availability

The corresponding author will provide the datasets created and/or analyzed during the current study upon reasonable request.
