# Supplementary material for: A dual methodology employing ion-pair chromatography and built-in UV spectrophotometry for quantifying recently approved combination of mometasone and indacaterol in a novel combined metered dose inhaler: assessing the greenness, carbon footprint, blueness, and whiteness
Source: BMC Chem. 2024 Aug 3;18(1):143. doi: 10.1186/s13065-024-01242-y (PMC11297607; doi:10.1186/s13065-024-01242-y)
Supplement: Supplementary file 1 — Supplementary Material 1. [file 13065_2024_1242_MOESM1_ESM.docx]

**Supplementary Tables**

**Table S1.** Optimization of chromatographic conditions for the determination of studied drugs by the proposed IPC method

|  | | **No of theoretical plates (N)** | | **Resolution (R_s_)** | **Tailing factor (T)** | |
| --- | --- | --- | --- | --- | --- | --- |
|  |  | **IND** | **MOM** | **IND/MOM** | **IND** | **MOM** |
| **pH** | 2.5 | 1550 | 2122 | 5.52 | 1.11 | 1.07 |
|  | 3 | 1521 | 2172 | 5.25 | 1.01 | 1.11 |
|  | 4 | 1495 | 1601 | 4.35 | 1.33 | 1.18 |
|  | 5 | 1418 | 1552 | 3.82 | 1.88 | 1.44 |
|  | 6 | 1385 | 1473 | 3.13 | 2.29 | 1.64 |
| **Acetonitrile:**  **acidified water containing 0.025% SDS (v/v)** | 40:60% | 527 | 576 | 1.33 | 1.24 | 1.26 |
|  | 45:55% | 355 | 469 | 1.56 | 1.16 | 1.12 |
|  | 50:50% | 1521 | 2172 | 5.25 | 1.01 | 1.11 |
|  | 55:45% | 1114 | 1777 | 5.07 | 1.18 | 1.11 |
|  | 60:40 % | 694 | 1284 | 4.66 | 0.67 | 0.91 |
| **The ionic strength of OPA (M)** | 0.05 | 1507 | 2151 | 5.27 | 1.12 | 1.01 |
|  | 0.1 | 1521 | 2172 | 5.25 | 1.01 | 1.11 |
|  | 0.15 | 1311 | 2085 | 5.70 | 1.24 | 1.14 |
|  | 0.2 | 1062 | 1865 | 5.67 | 2.17 | 1.54 |
|  | 0.3 | 738 | 1331 | 4.85 | 1.44 | 1.25 |
| **Flow rate (mL/min)** | 0.8 | 1312 | 2198 | 5.14 | 2.25 | 1.91 |
|  | **1** | 1521 | 2172 | 5.25 | 1.01 | 1.11 |
|  | 1.2 | 1157 | 1068 | 5.71 | 0.96 | 0.98 |

- Where: Number of theoretical plates (N) = 5.45(t_R_/ W_h/2_)^2^.
- W_h/2_ is the half peak width.
- Resolution (R_s_) = 2 ∆ t_R_/W_1_ +W_2_.
- W_1_ and W_2_ are the peak widths of the two components measured at their bases.
- t_R_ is the time of retention of the measured substance measured from the injection point and t_M_ is the retention time of a non-retained marker.

**Table S2: Determination of IND and MOM in laboratory-prepared mixtures by the proposed methods.**

| **Lab prepared mixture**  **(µg mL^-1^)** | | **IPC method**  **(Recovery %^a^)** | | **Spectroscopic methods (Recovery %^a^)** | | | |
| --- | --- | --- | --- | --- | --- | --- | --- |
|  |  |  |  | **First derivative (^1^D)** | | **Ratio derivative (RD)** | |
| **MOM** | **IND** | **MOM** | **IND** | **MOM** | **IND** | **MOM** | **IND** |
| 1.6 | 1.5 | 100.32 | 101.21 | 101.62 | 100.58 | 98.82 | 99.40 |
| 3.2 | 3 | 98.20 | 99.54 | 101.40 | 99.74 | 102.05 | 100.90 |
| 4.8 | 4.5 | 101.02 | 99.76 | 98.15 | 98.45 | 100.76 | 99.61 |
| 6.4 | 6 | 98.26 | 100.65 | 101.30 | 99.41 | 98.93 | 100.09 |
| 8 | 7.5 | 98.34 | 100.87 | 98.72 | 100.64 | 101.01 | 101.00 |
| 9.6 | 9 | 99.32 | 100.89 | 98.65 | 101.39 | 100.48 | 99.34 |
| 2 | 2 | 99.32 | 100.89 | 98.65 | 101.39 | 100.48 | 99.34 |
| 2 | 4 | 100.29 | 101.18 | 101.59 | 100.55 | 98.79 | 99.37 |
| 2 | 6 | 98.17 | 99.51 | 101.37 | 99.71 | 102.02 | 100.87 |
| 6 | 2 | 100.99 | 99.73 | 98.12 | 98.42 | 100.73 | 99.58 |
| 8 | 2 | 98.23 | 100.62 | 101.27 | 99.38 | 98.9 | 100.06 |
| 7.5 | 1.5 | 98.31 | 100.84 | 98.69 | 100.61 | 100.98 | 100.97 |
| **Mean %**  **± RSD** | | 99.23  ±1.153 | 100.47  **±**0.643 | 99.96  ±1.545 | 100.02  ±1.003 | 100.33  ±1.192 | 100.04  ±0.704 |

^a^ Each result is the average of three separate determinations.

**Table S3: Statistical comparison of the results obtained by the proposed methods and the reported method for the analysis of IND and MOM**

| **Parameters** | **IPC method** | | **Spectroscopic methods** | | | | **Reported method** (Bahgat et al., 2023) | |
| --- | --- | --- | --- | --- | --- | --- | --- | --- |
|  |  |  | **First derivative (^1^D)** | | **Ratio derivative (RD)** | |  |  |
|  | **MOM** | **IND** | **MOM** | **IND** | **MOM** | **IND** | **MOM** | **IND** |
| n^a^ | 5 | 5 | 5 | 5 | 5 | 5 | 5 | 5 |
| Mean % | 100.12 | 98.96 | 100.07 | 100.31 | 100.17 | 99.90 | 100.17 | 99.64 |
| SD | 0.392 | 0.972 | 0.391 | 1.019 | 0.396 | 0.839 | 0.268 | 0.909 |
| variance | 0.153 | 0.944 | 0.153 | 1.038 | 0.157 | 0.705 | 0.072 | 0.826 |
| Student’s t-test ^b^  (2.306) | 0.227 | 1.200 | 0.456 | 1.046 | 0.019 | 0.416 | — | — |
| F-value ^b^  (6.388) | 2.125 | 1.144 | 2.125 | 1.257 | 2.181 | 1.172 | — | — |

^a^ Number of experiments.

^b^ The values in parenthesis are tabulated values of “t “and “F” at (P = 0.05).

|  | **Proposed IPC Method** | **Reported Method**  (Bahgat et al., 2023) | **Reported Method**  (Youssef et al., 2023) | **Reported Method**  (Tarek et al., 2023) | **Proposed Spectroscopic methods** |
| --- | --- | --- | --- | --- | --- |
| **Type of analysis** | Quantitative and confirmatory | Quantitative and confirmatory | Quantitative and confirmatory | Quantitative and confirmatory | Quantitative and confirmatory |
| **Multi or single-element analysis** | 2-15 analyses of different chemical class | 2-15 analyses of different chemical class | 2-15 analyses of different chemical class | 2-15 analyses of different chemical class | 2-15 analyses of different chemical class |
| **Analytical technique** | IPC Method | Micellar liquid chromatographic method | RP-HPLC | RP-UPLC | Ultraviolet spectrometry |
| **Simultaneous sample preparation** | Simultaneous sample preparation of 2-12 samples | Simultaneous sample preparation of 2-12 samples | Simultaneous sample preparation of 2-12 samples | Simultaneous sample preparation of 2-12 samples | Simultaneous sample preparation of 2-12 samples |
| **sample preparation** | No sample preparation is Required | No sample preparation is Required | No sample preparation is Required | No sample preparation is Required | No sample preparation is Required |
| **sample per hour** | 15 | 7.5 | 5 | 6 | 60 |
| **Reagents and materials** | SDS (0.025%) and acetonitrile | SDS  (191.49 mM), methanol, and potassium dihydrogen phosphate buffer | Methanol: 0.1% glacial acetic acid | Methanol, and formic acid | Water and ethanol |
| **Preconcentration** | No pre-concentration is needed | No pre-concentration is needed | No pre-concentration is needed | No pre-concentration is needed | No pre-concentration is needed |
| **Degree of automation** | Semi-automated methods with common devices | Semi-automated methods with common devices | Semi-automated methods with common devices | Semi-automated methods with common devices | Semi-automated methods with common devices |
| **Amount of sample** | Sample volumes below 100 μL | Sample volumes below 100 μL | Sample volumes below 100 μL | Sample volumes below 100 μL | Sample volumes below 100 μL |

**Table S4: Statistical comparison of the results obtained by the proposed methods and the reported method for the analysis of IND and MOM**

**Supplementary Figure**

**MOM**

**IND**

**
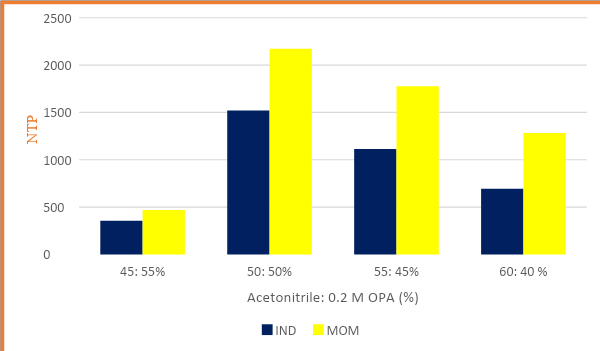
**

**Fig. S1.** Effect of different % mobile phase ratios on the number of theoretical plates of IND 7.5 µg mL^-1^, and MOM 8 µg mL^-1^ using a mobile phase consisting of acetonitrile and acidified water containing 0.025% sodium dodecyl sulfate using 0.1 M OPA at pH 3.0 and flow rate, 1.0 mL/min.

**
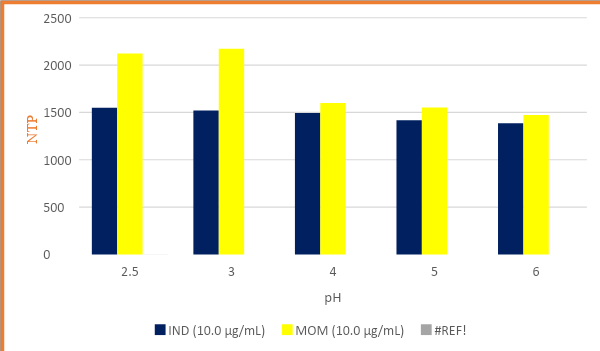
**

**MOM**

**IND**

**Fig. S2.** Effect of different pH on the number of theoretical plates of IND 7.5 µg mL^-1^, and MOM 8 µg mL^-1^ using a mobile phase consisting of acetonitrile and acidified water containing 0.025% sodium dodecyl sulfate (50: 50% v/v) using 0.1 M OPA and flow rate, 1.0 mL/min.

**
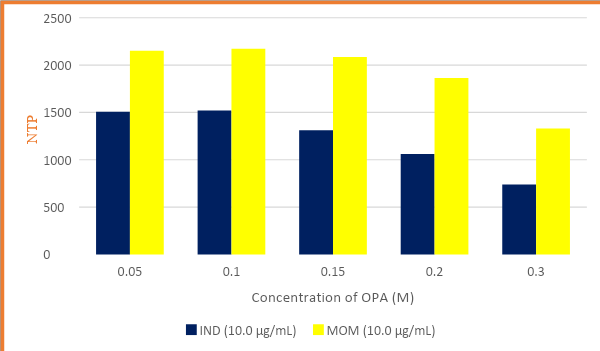
**

**MOM**

**IND**

**Fig. S3.** Effect of the different molar concentrations of OPA on the number of theoretical plates of IND 7.5 µg mL^-1^, and MOM 8 µg mL^-1^ using a mobile phase consisting of acetonitrile and acidified water containing 0.025% sodium dodecyl sulfate (50: 50% v/v) at pH 3.0 and flow rate, 1.0 mL/min.

**
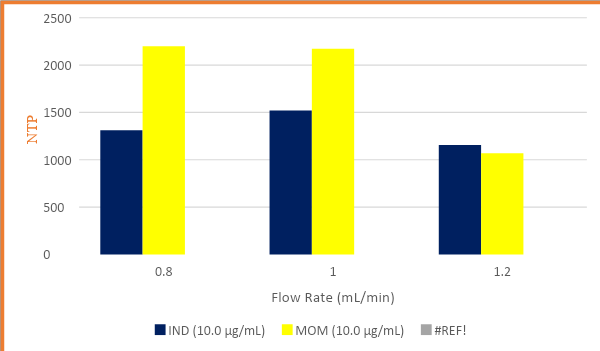
**

**MOM**

**IND**

**Fig. S4.** Effect of different flowrate on the number of theoretical plates of IND 7.5 µg mL^-1^, and MOM 8 µg mL^-1^ using a mobile phase consisting of acetonitrile and acidified water containing 0.025% sodium dodecyl sulfate (50: 50% v/v) using 0.1 M OPA at pH 3.0.


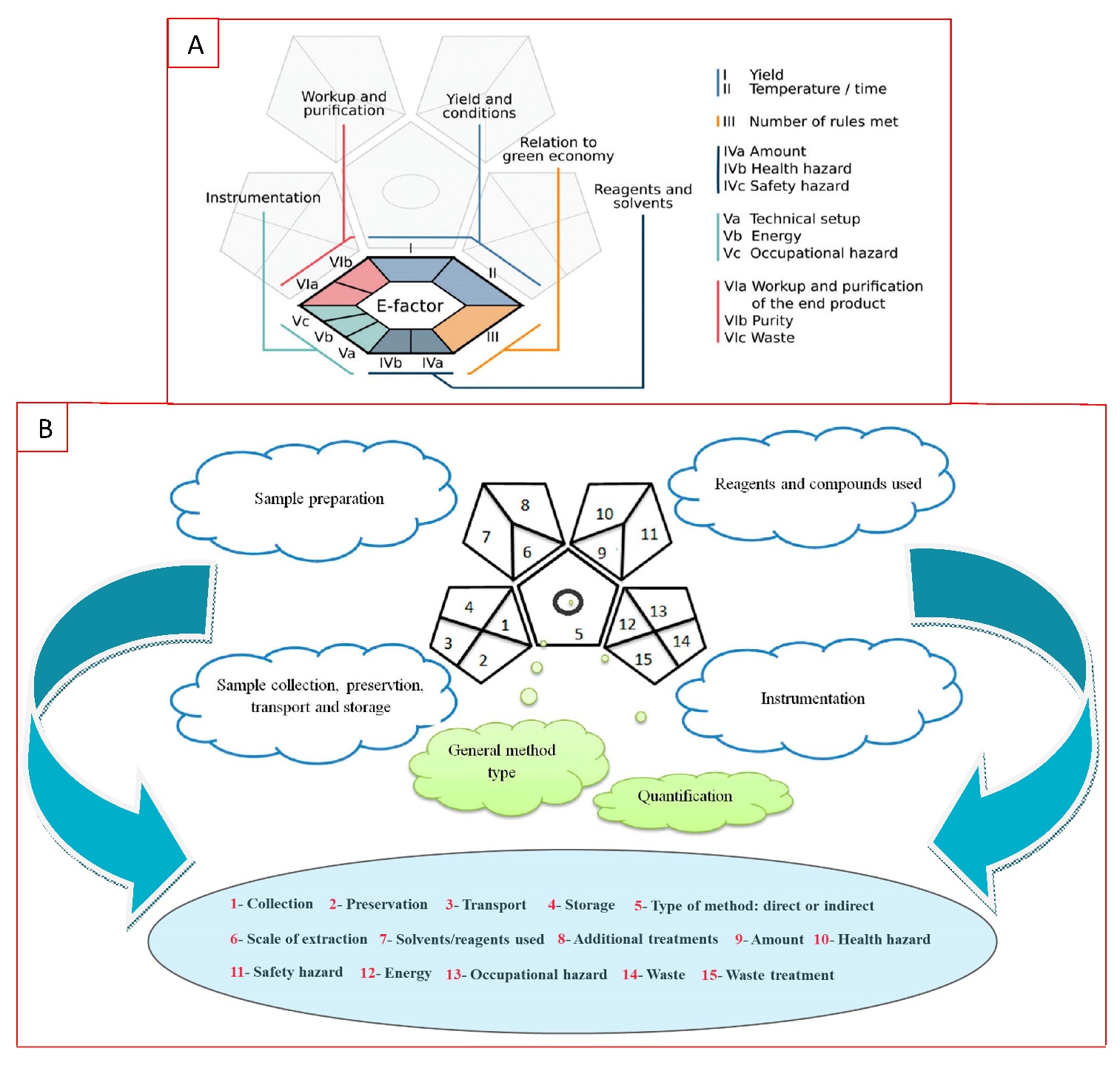


**Fig. S5.** (A) The ComplexGAPI pictogram, with the original GAPI pictogram greyed out in the background, and particular fields of the added hexagonal glyph grouped and color-coded for clarity. (B) 15 zones in the original GAPI pictogram together with a brief explanation of the assessed parameter by each zone

**
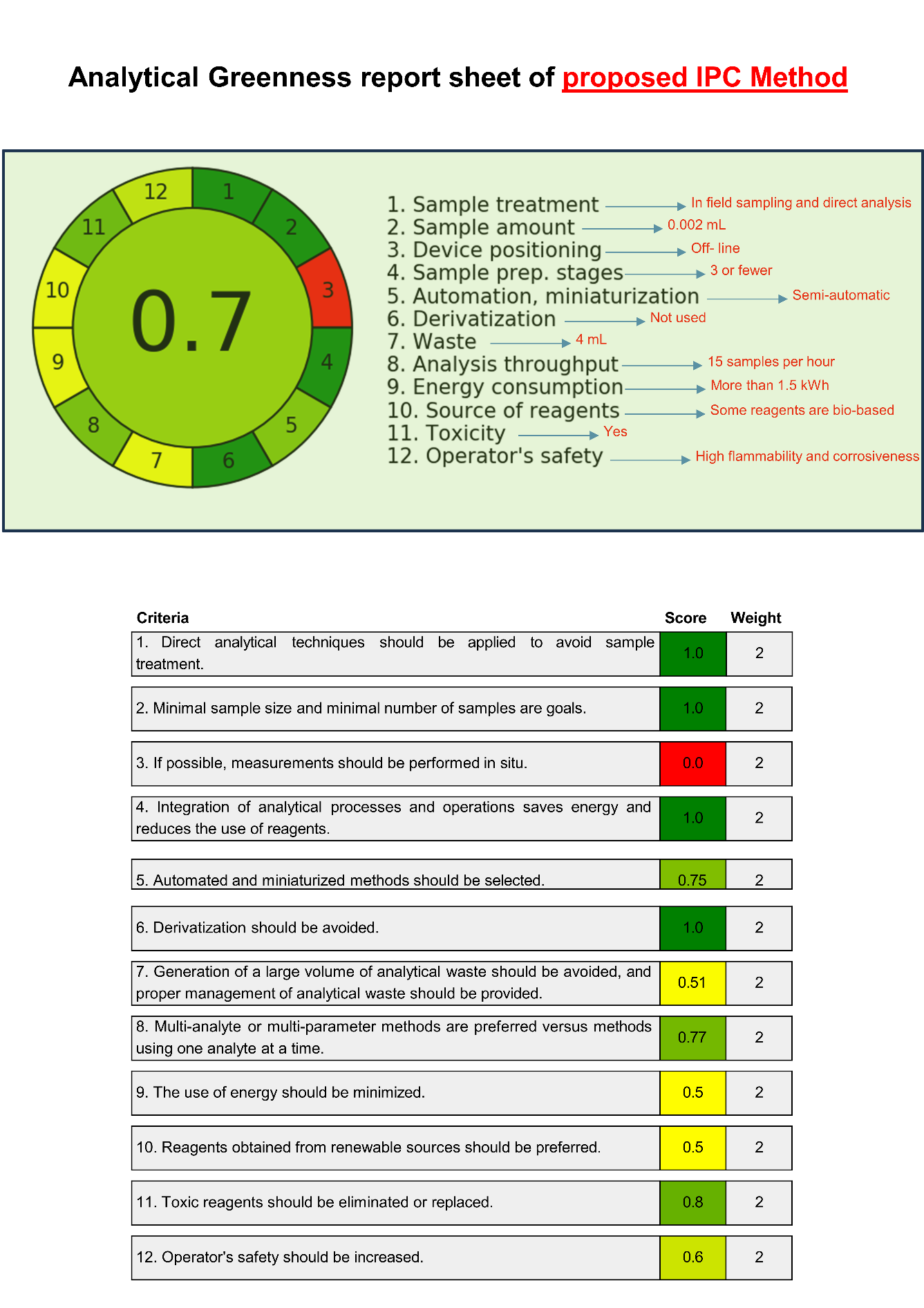
**

**Fig. S6.** Analytical greenness report sheets for the **proposed IPC** **method** by AGREE tool comprising the input data of AGREE calculator.

**
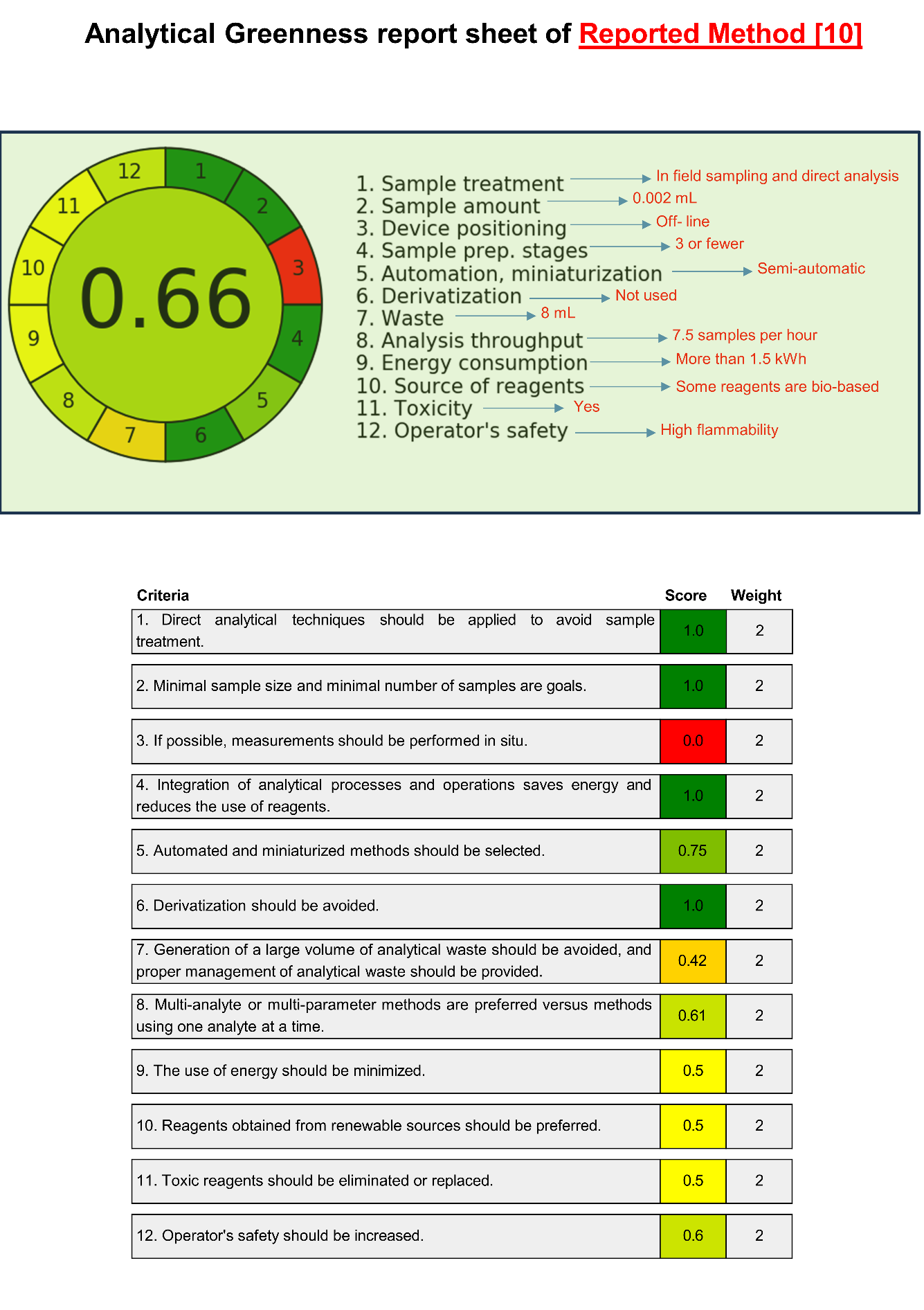
**

**Fig. S7.** Analytical greenness report sheets for the **reported** **method [10]** by AGREE tool comprising the input data of AGREE calculator.

**
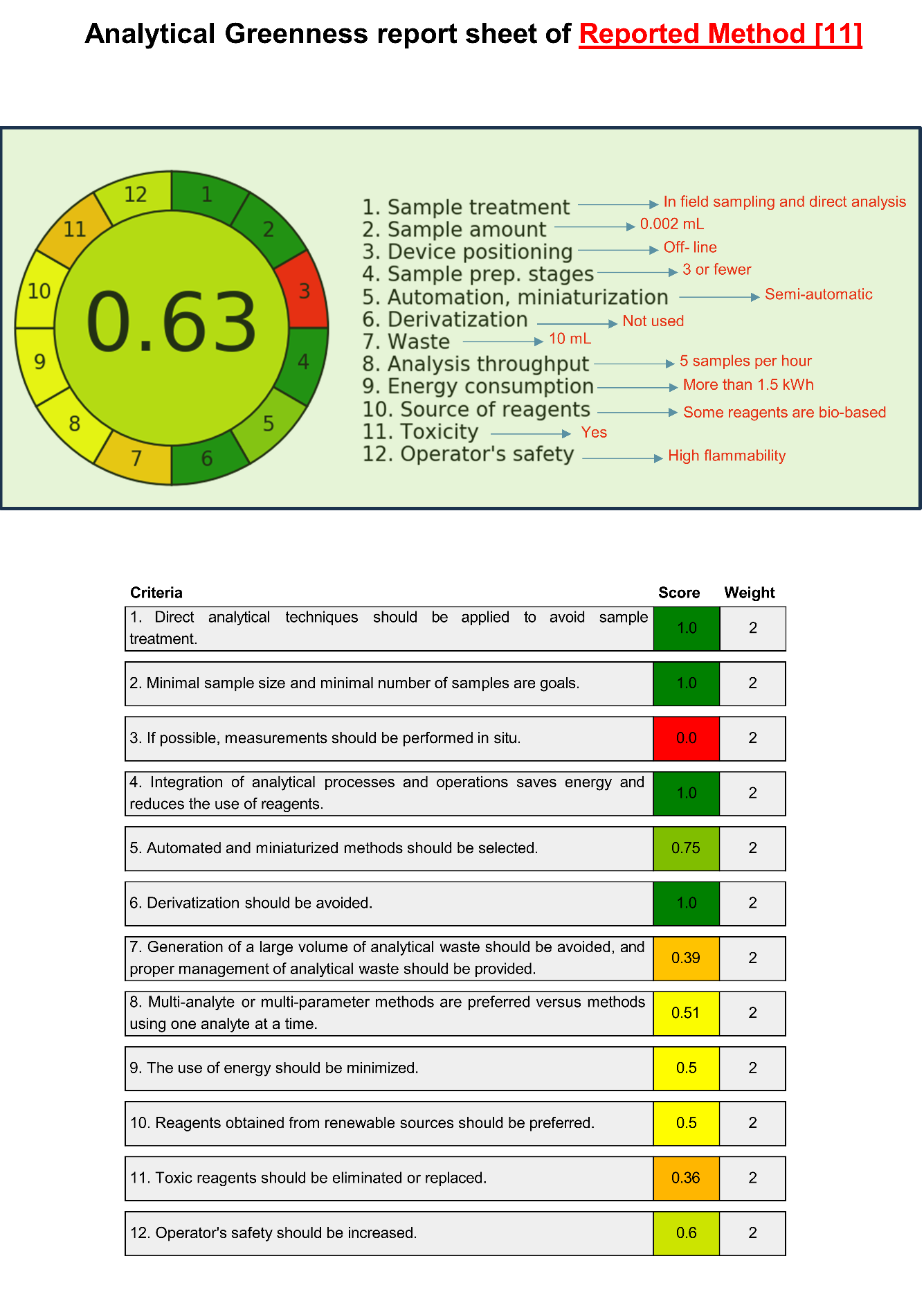
**

**Fig. S8.** Analytical greenness report sheets for the **reported** **method [11]** by AGREE tool comprising the input data of AGREE calculator.

**
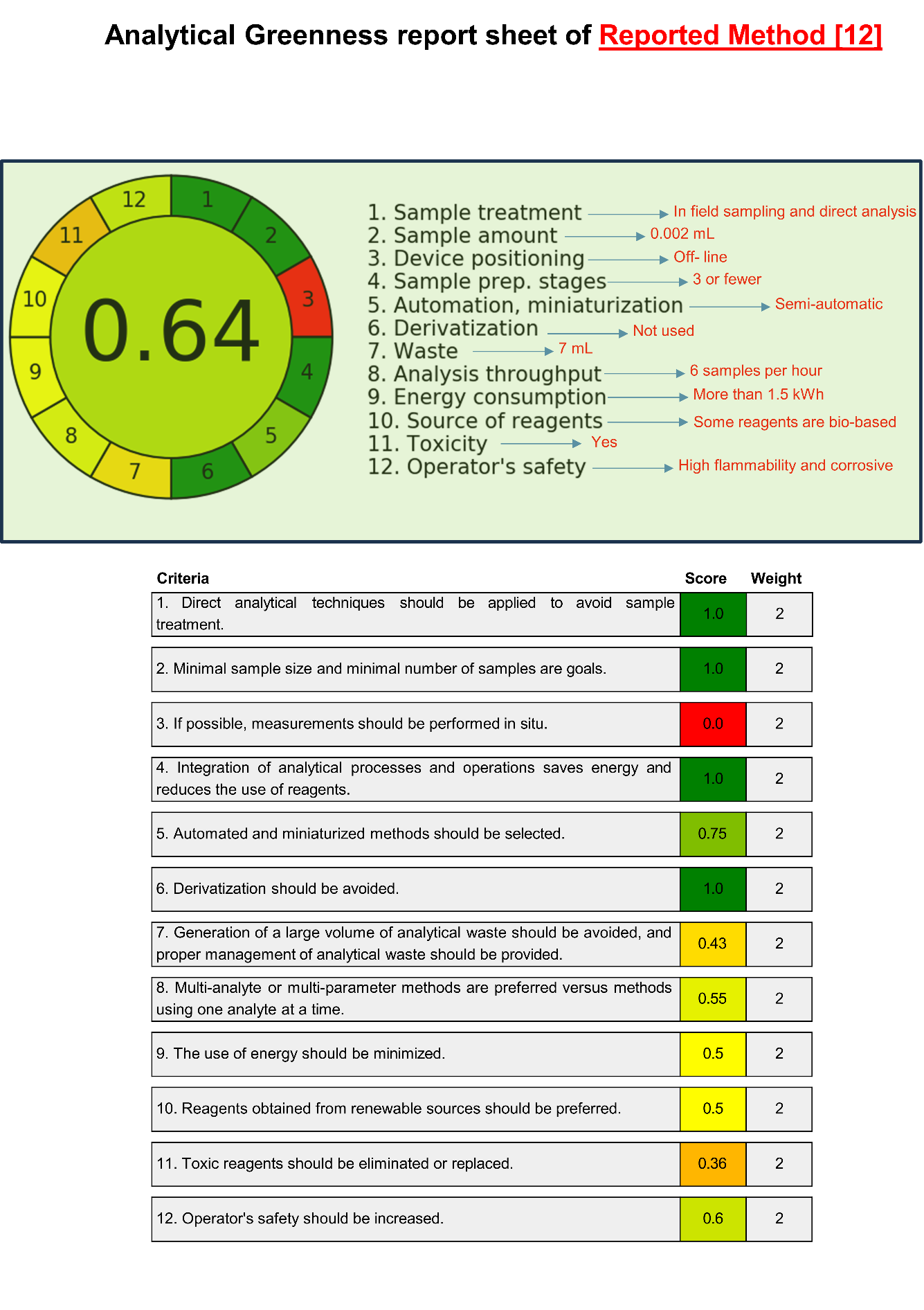
**

**Fig. S9.** Analytical greenness report sheets for the **reported** **method [12]** by AGREE tool comprising the input data of AGREE calculator.


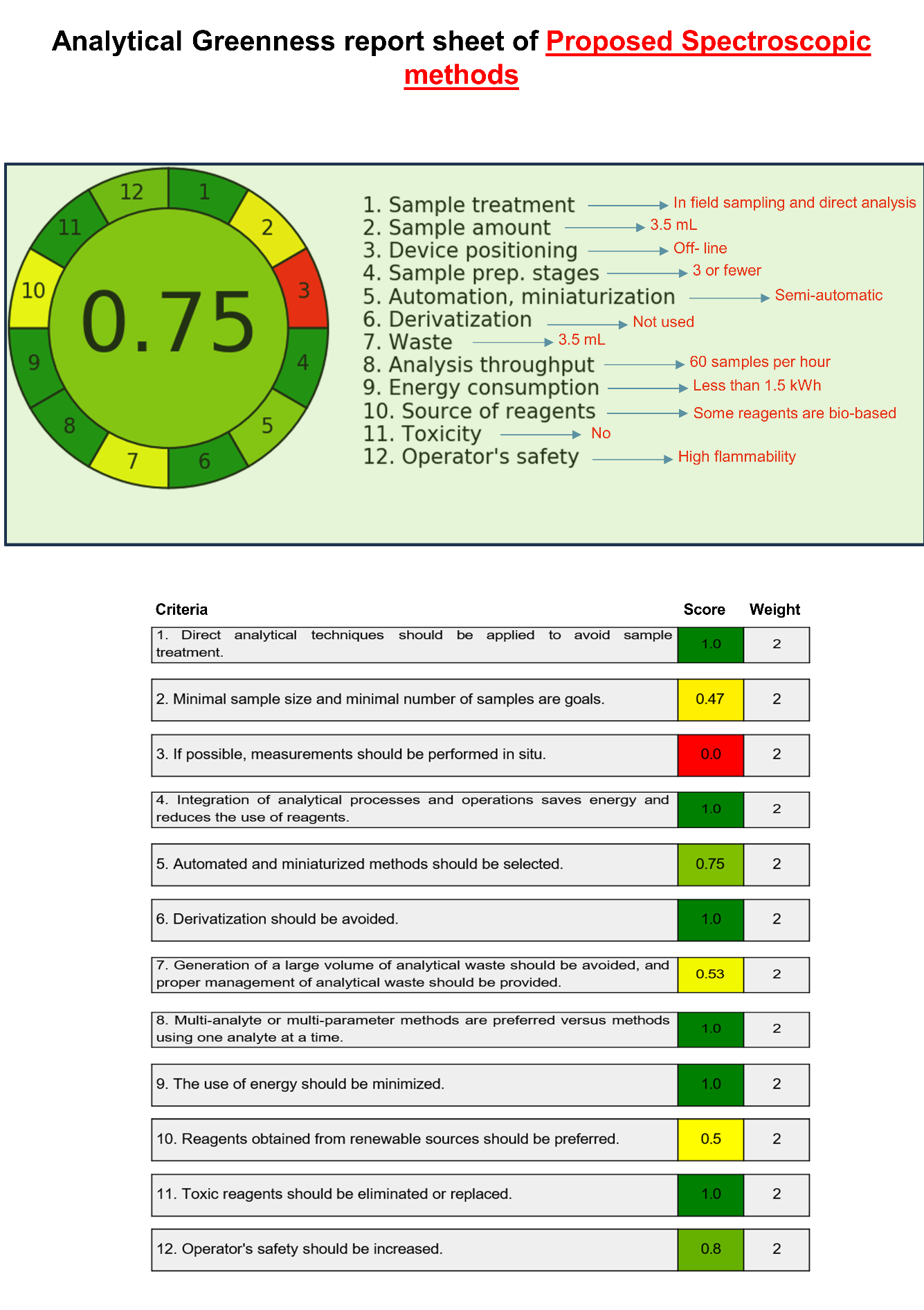


**Fig. S10.** Analytical greenness report sheets for the **proposed spectroscopic** **methods** by AGREE tool comprising the input data of AGREE calculator.


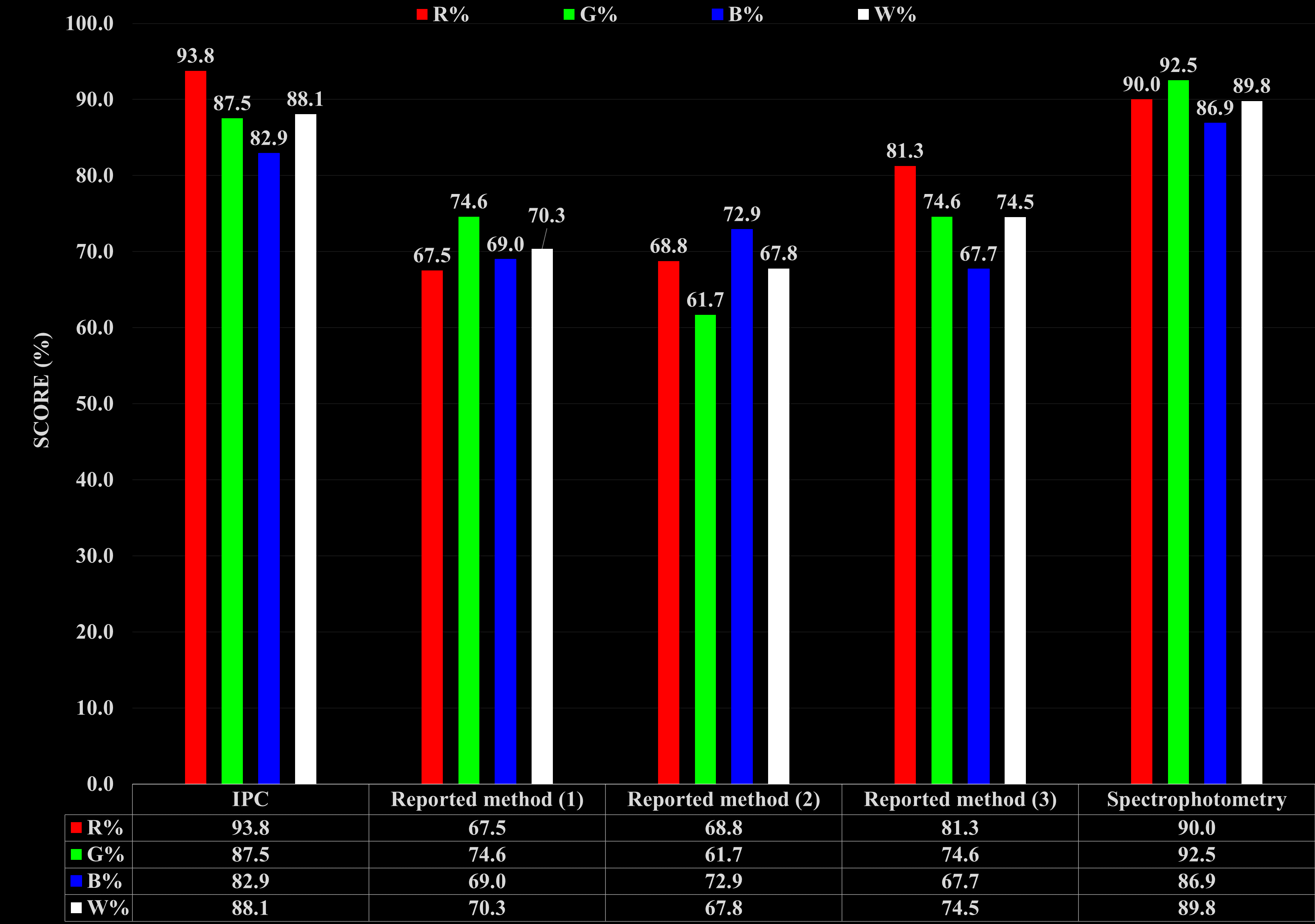


**Fig. S11.** Comparison of the redness, greenness, blueness, and whiteness profiles of the proposed and reported methods, obtained by the RGB 12 algorithm.
